# Regulation of carcinogenesis and modulation through Wnt/β-catenin signaling by curcumin in an ovarian cancer cell line

**DOI:** 10.1038/s41598-019-53509-3

**Published:** 2019-11-21

**Authors:** Hsing-Yu Yen, Chih-Wei Tsao, Ya-Wen Lin, Chih-Chi Kuo, Chang-Huei Tsao, Chin-Yu Liu

**Affiliations:** 1 Department of Nutritional Science, Fu Jen Catholic University, Taipei, Taiwan; 2 Division of Urology, Department of Surgery, Tri-Service General Hospital, National Defense Medical Center, Taipei, Taiwan; 3 Department of Microbiology and Immunology, National Defense Medical Centre, Taipei, Taiwan; 4 Teaching and Research Office, Tri-Service General Hospital Songshan Branch, Taipei, Taiwan

**Keywords:** Ovarian cancer, Ovarian cancer, Cancer epigenetics, Cancer epigenetics, DNA methylation

## Abstract

The secreted frizzled-related protein 5 gene *(SFRP5)* that antagonize the Wnt/β-catenin signaling is frequently inactivated by promoter methylation and oncogenic activation of the Wnt signaling pathway is common in many cancers. The curcumin-rich *Curcuma longa* has been reported to potent anti-cancer property involved in epigenetic regulation to inhibit tumor suppressor gene methylation and re-expression. In a compounds screening, we found that curcumin can inhibit Wnt/β-catenin signaling. Therefore, the aim of this study was to investigate the effects of curcumin on SFRP5 DNA methylation modification in an ovarian cancer cell line (SKOV3). SKOV3 cells were treated with DMSO, 10 μM 5-aza-2′-deoxycytidine (DAC), 5 μM DAC, 20 μM curcumin, and 20 μM curcumin combined with 5 μM DAC for 96 hours, following which RNA and proteins were extracted for further analysis. The results showed that curcumin combined with 5 μM DAC may inhibit cancer cell colony formation, migration through EMT (epithelial–mesenchymal transition) process regulation, total DNMT activity, especially in DNMT3a protein expression, and may also regulate tumor suppressor gene SFRP5 expression involved in the Wnt/β-catenin signaling pathway. The combined treatment attenuated ovarian cancer development.

## Introduction

Ovarian cancer, one of the ten common and leading causes of cancer death among women worldwide, is the fifth and seventh leading cause of death from cancer in females in the United States and Taiwan, respectively^1–3^. It is a major cause of gynecological cancer-associated mortality^4^. Currently, one difficulty in identifying ovarian cancer is that it presents with vague, non-specific symptoms, including abdominal pain, pelvic pain and constipation, and the lack of reliable diagnostic biomarkers results in women often being diagnosed with advanced-stage ovarian cancer^5–7^. The later the phase in which cancer is diagnosed, the poorer the prognosis, and the higher the rates of relapse, invasion, metastasis and chemo-resistance^8^; these complications involve the EMT process, which may be regulated by the Wnt/β-catenin signaling pathway^9,10^.

The Wnt signaling pathway, an essential pathway in embryogenesis, plays a key role in cancer development^11^. Aberrant Wnt signaling is present in many cancers, including breast, colorectal, prostate and ovarian cancers^12^. It is composed of three different molecular pathways, one of which is the canonical pathway, also called the Wnt/β-catenin signaling pathway, which is dependent on a stable β-catenin structure; target genes may lead to proliferation and EMT in cancer cells^13^. Aberrant Wnt and β-catenin expressions are common in ovarian cancer^14,15^. Stabilization of β-catenin by Wnt signaling allows β-catenin translocation into the nucleus as a cofactor of the T-cell factor (TCF)/lymphoid enhancer factor (LEF) family of transcription factors, and triggers the expressions of downstream genes, such as c-Myc, Cyclin D1, Axin, Survivin, and matrix metalloproteinases^16^.

Previous research indicated that many components of the Wnt/β-catenin signaling pathway may be regarded as targets in cancer therapy and could prevent cancerous aggravation^17^. The secreted frizzled-related protein (SFRP) family consists of tumor suppressor genes including five secreted glycoproteins with Wnt-binding properties of the cysteine-rich domain^18^. SFRPs are inhibitors of the Wnt signaling pathway; however, studies have indicated that SFRPs as Wnt antagonist genes are often silenced owing to promoter region hypermethylation in cancers such as bladder, breast, head and neck, and colorectal cancers^19–21^. Hypermethylation of SFRP1, SFRP2 and SFRP5 in ovarian cancer in Taiwanese women is a common phenomenon^22^. SFRP5, an independent risk factor for overall survival in patients with ovarian cancer, is hypermethylated alongside tumor growth and chemosensitivity in ovarian cancer^23^.

DNA methylation occurs at the fifth carbon position of cytosine residues within CpG islands by enzymes called DNA methyltransferases (DNMTs) through transfer of the methyl group from S-adenosylmethionine (SAM)^24^. The DNMT family consists of DNMT1, DNMT3a and DNMT3b. DNMT1, as a maintenance enzyme, binds and transfers the methyl group to hemi-methylated DNA during replication. DNMT3a and DNMT3b, as *de novo* enzymes, bind and add a methyl group to un-methylated DNA^25^. DNMT inhibitors can obstruct DNA methylation, resulting in gene re-expression, represented by hypermethylation in cancers. One DNMT inhibitor, 5-aza-2′-deoxycytidine (DAC), is an antitumor agent approved by the FDA for the treatment of myelodysplastic syndrome (MDS)^26^. DAC, a cytidine analog containing a nitrogen atom, is also called decitabine in place of cytidine during DNA replication, whereupon it forms covalent bonds with DNMTs, leading to inactivation. However, DAC forms DNMT–DNA adducts with dose-dependent toxicity^27^. In addition, there are some of side effects of treatment with DAC for MDS and lung cancer, for example, neutropenia, thrombocytopenia and anemia^28^. In the light of this, lower doses are prescribed to minimize the toxicity of DAC; otherwise, improvement of the chemosensitivity of cancer cells is necessary.

For ovarian cancer, chemotherapy is usually a combined treatment that involves at least two different types of chemo drugs together. Although tumors often shrinks or go away with the treatment, cancer cells are eventually resistant to the drugs and grow again. The progress of new drug development is in urgent need and is an ongoing work. Instead of new drug development, various natural products are now found to have their pharmacological effects and the potential in serving as effective substances against drug resistance is believed^29^. Additionally, the effects of natural products (such as curcumin) on DNA methylation in cancer cells are also showed in current studies^30,31^. However, the impacts of combined natural compounds and DAC on improvement of the chemosensitivity or reduction of the chemoresistance of cancer cells are limited.

Curcumin (diferuloylmethane) is a yellow pigment of natural polyphenol derived from the rhizome of *Curcuma longa*, also called turmeric^32^. Turmeric contains several compounds, including curcuminoids, which are comprised of curcumin, demethoxycurcumin and bisdemethoxycurcumin^33^. Curcumin has long been used as a traditional therapy in the same way as herbal aspirin in India. In addition, curcumin exerts anti-oxidant, anti-inflammatory and anti-cancer effects, including angiogenesis and cell-cycle inhibition^34^. Previous research has indicated that curcumin has been used in single and combination treatments for many cancers, such as breast, colorectal, oral and prostate cancers^35^. It is also involved in regulating the Wnt/β-catenin signaling pathway in bladder, gastric and lung cancer cells^36–38^. However, the effects of curcumin on SFRP5 regulation are not clear. The aim of current study was to investigate the effects of single or combination treatment with curcumin and DAC on DNA methylation modification of SFRP5 and the Wnt/β-catenin signaling pathway in ovarian cancer.

## Results

### Effects of curcumin on SKOV3 cell viability

**Figure 1:**
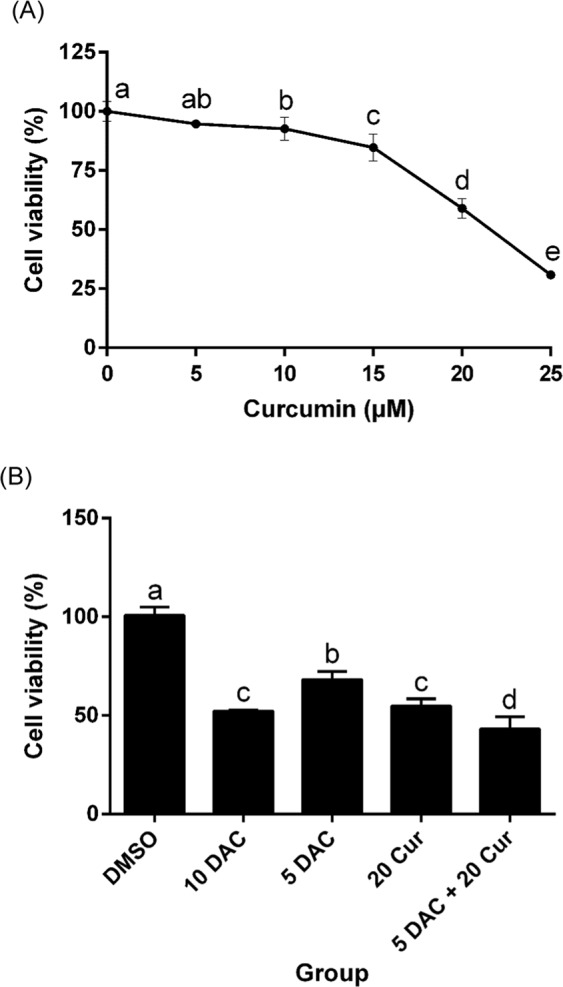
Effects of curcumin on SKOV3 cell viability. SKOV3 cells treated with curcumin alone (**A**) and combined with DAC (**B**). 10 DAC, 10 μM DAC; 5 DAC, 5 μM DAC; 20 Cur, 20 μM curcumin for 96 hours. Data are expressed as means ± SD of triplicate experiments. ^a,b,c,d^Points or bars without the same letters on top are statistically significant among treatments when compared to each other, as determined by one-way ANOVA followed by Duncan’s *post hoc* test (*p* < 0.05).

SKOV3 cells were treated with curcumin, DAC, and a combination of both for 96 hours at different concentrations. The cell viability was analyzed using an MTS assay. Curcumin suppressed the proliferation of SKOV3 cells, the IC_50_ of curcumin being approximately 20 μM (Fig. 1A). The inhibition effect on cell growth of combined treatment with 5 μM DAC and 20 μM curcumin was superior to the effects of single treatments (Fig. 1B).

### Effects of curcumin combined with DAC on the methylation status and mRNA expression of SFRP5

**Figure 2:**
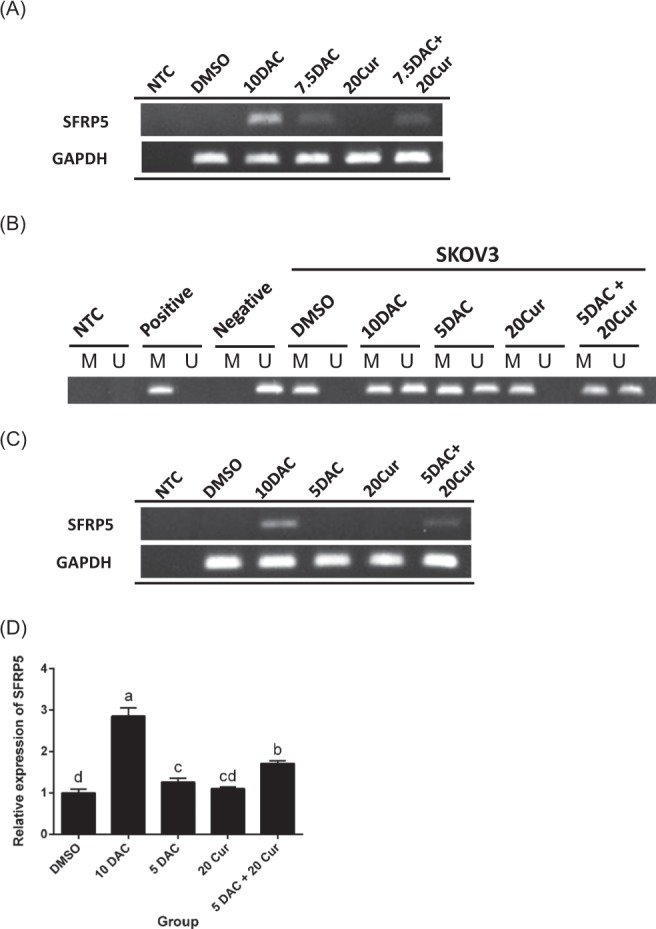
Effects of curcumin alone and in combination with DAC for 96 hours on the methylation status and mRNA expression of SFRP5 in SKOV3 ovarian cancer cells. (**A**,**C**) RT-PCR of SFRP5 gene expression. (**B**) MS-PCR of SFRP5 gene methylation status. (**D**) Q-PCR of SFRP5 gene expression level. 10 DAC, 10 μM DAC; 5 DAC, 5 μM DAC; 20 Cur, 20 μM curcumin; NTC, no template control; Positive: CpG methylated human genomic DNA (positive control); Negative: DNA of peripheral blood lymphocytes (negative control); M: methylated status; U: un-methylated status. Data are expressed as means ± SD of triplicate experiments. ^a,b,c,d^Bars without the same letters on top are statistically significant among treatments when compared to each other, as determined by one-way ANOVA followed by Duncan’s *post hoc* test (*p* < 0.05).

We conducted an investigation of SFRP5 expression variation upon combined treatment with 7.5 μM or 5 μM DAC and 20 μM curcumin using RT-PCR, and found that 5 μM DAC and 20 μM curcumin obviously changed the expression from no signal band to a slight signal band (Fig. 2A,C). The MS-PCR data showed that SFRP5 was partially converted to an un-methylated status under combined treated with 5 μM DAC and 20 μM curcumin (Fig. 2B). We further examined the mRNA expression levels by Q-PCR, and found that SFRP5 was up-regulated under combined treatment, in contrast to single treatment with 5 μM DAC or 20 μM curcumin (Fig. 2D). 10 μM DAC was used as a positive control for the de-methylation and re-expression of SFRP5.

### Effects of curcumin alone and in combination with DAC on colony formation in SKOV3 ovarian cancer cells

**Figure 3:**
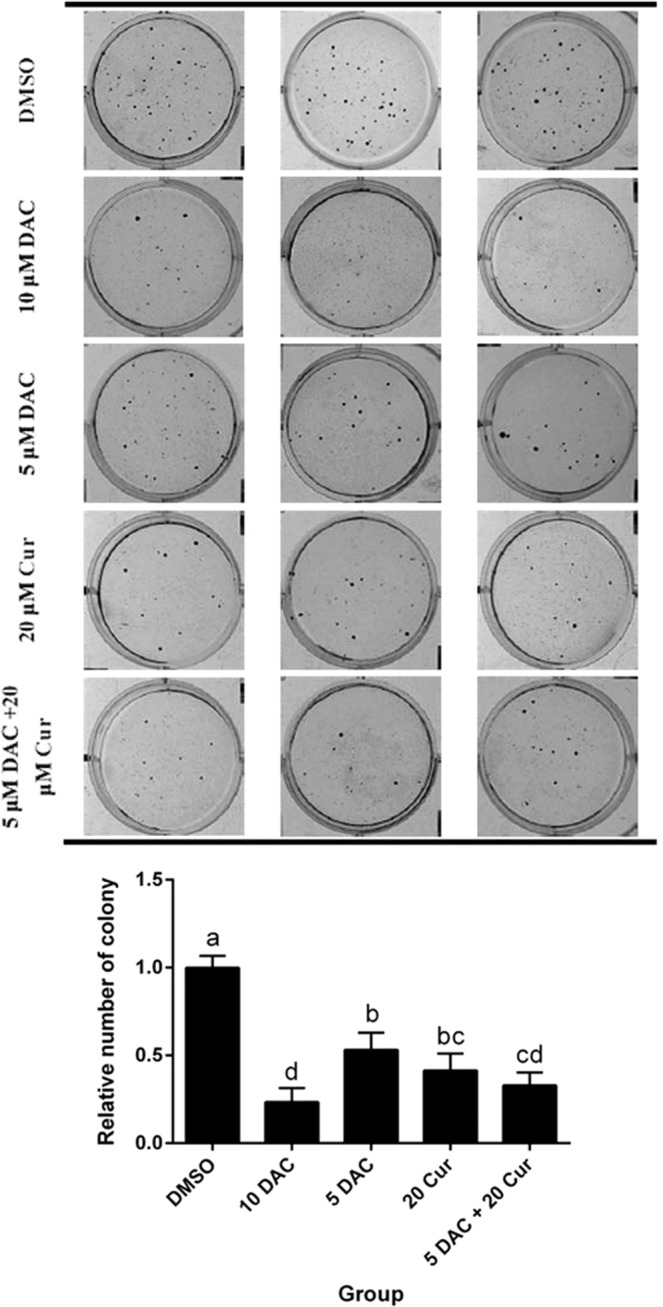
Effects of curcumin alone and in combination with DAC for 96 hours on colony formation in SKOV3 ovarian cancer cells. 10 DAC, 10 μM DAC; 5 DAC, 5 μM DAC; 20 Cur, 20 μM curcumin. Data are expressed as means ± SD of triplicate experiments. ^a,b,c,d^Bars without the same letters on top are statistically significant among treatments when compared to each other, as determined by one-way ANOVA followed by Duncan’s *post hoc* test (*p* < 0.05).

The Wnt/β-catenin signaling pathway is involved in tumorigenesis. We investigated whether the compounds affected cell growth using a soft agar assay (also called an anchorage-independent growth assay). Both DAC and curcumin inhibited colony formation of SKOV3 cells. In addition, combined treatment with 5 μM DAC and 20 μM curcumin decreased the colony formation of SKOV3 cells by more than half (Fig. 3).

### Effects of curcumin alone and in combination with DAC on total DNMT activity and DNMT protein expression levels

**Figure 4:**
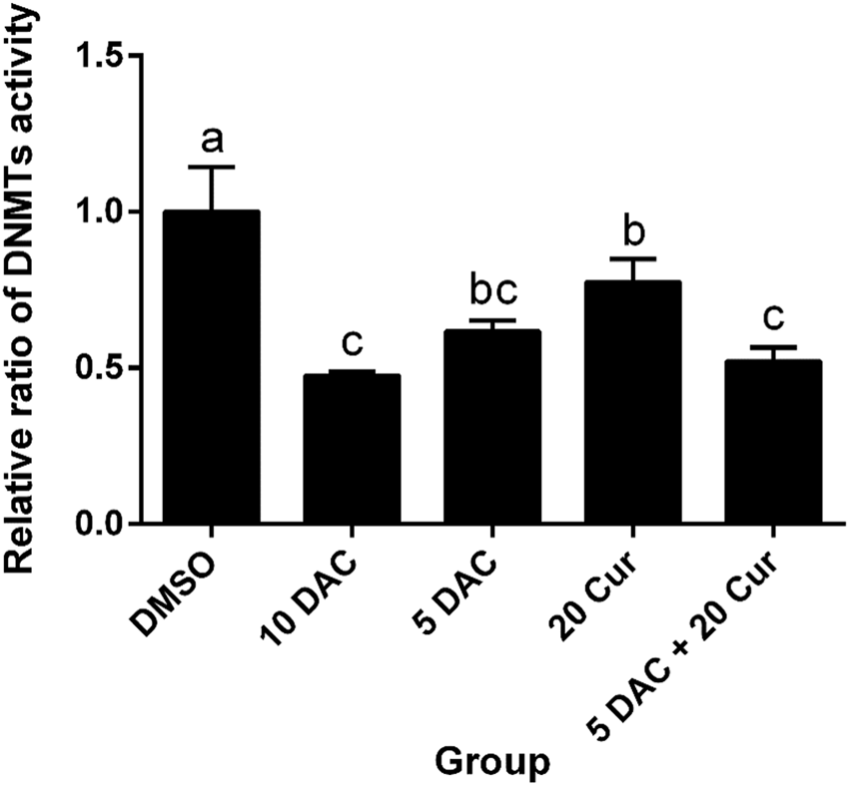
Effects of curcumin alone and in combination with DAC for 96 hours on total DNMT activity in SKOV3 ovarian cancer cells. 10 DAC, 10 μM DAC; 5 DAC, 5 μM DAC; 20 Cur, 20 μM curcumin. Data are expressed as means ± SD of triplicate experiments. ^a,b,c,d^Bars without the same letters on top are statistically significant among treatments when compared to each other, as determined by one-way ANOVA followed by Duncan’s *post hoc* test (*p* <0.05).

**Figure 5:**
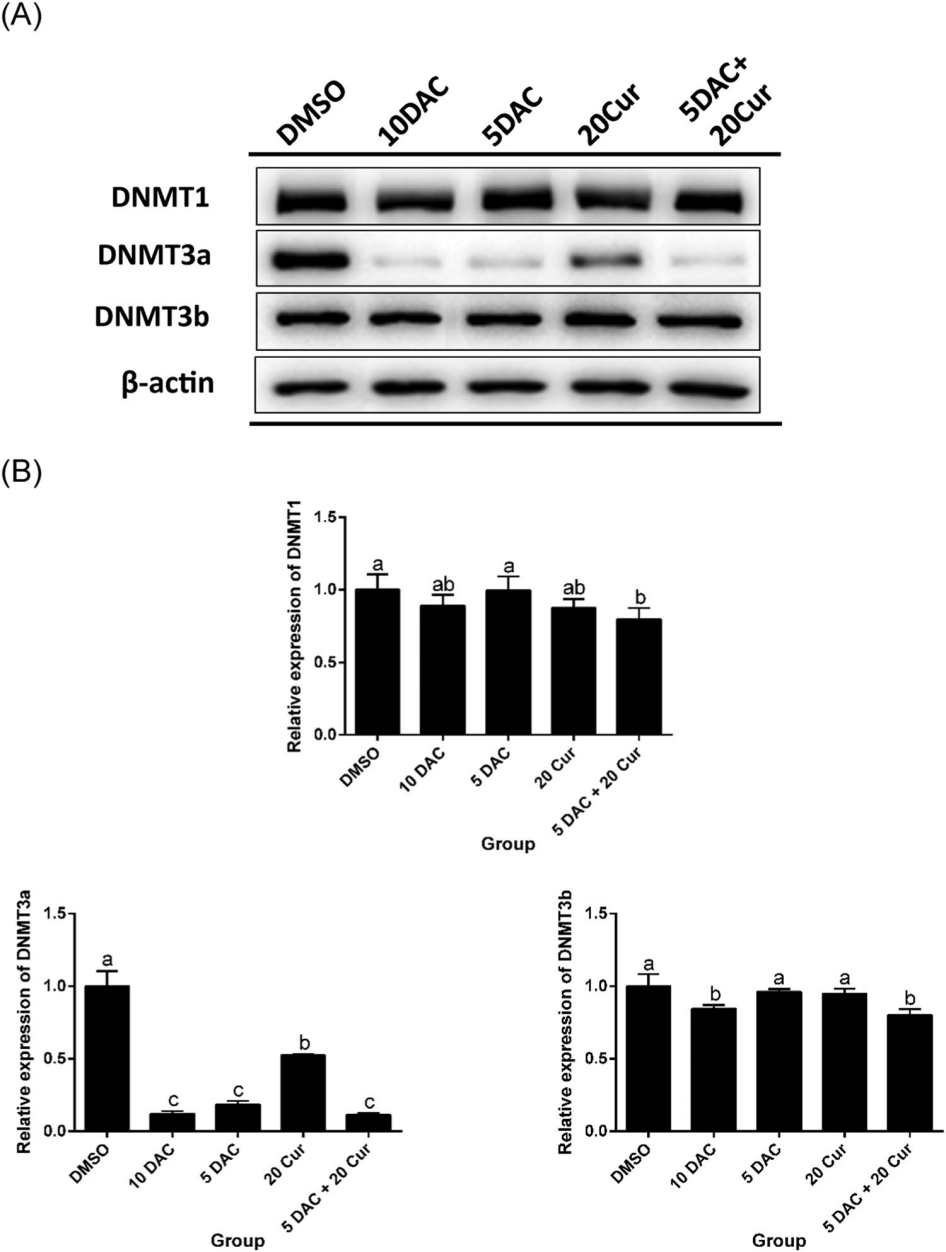
Effects of curcumin alone and in combination with DAC for 96 hours on DNMT protein expression levels in SKOV3 ovarian cancer cells. (**A**) Immunoblots for DNMT1, DNMT3a and DNMT3b proteins. (**B**) Densitometric analysis of DNMT1, DNMT3a and DNMT3b proteins. 10 DAC, 10 μM DAC; 5 DAC, 5 μM DAC; 20 Cur, 20 μM curcumin. Data are expressed as means ± SD of triplicate experiments. ^a,b,c,d^Bars without the same letters on top are statistically significant among treatments when compared to each other, as determined by one-way ANOVA followed by Duncan’s *post hoc* test (*p* <0.05).

The DNA methylation process occurs through DNMTs transferring a methyl group to DNA CpG islands. We further investigated whether the compounds affected the DNMT activity, decreasing the methylation status and increasing expression of SFRP5, in SKOV3 cells. We found that both DAC and curcumin inhibited the total activity of DNMTs. Combined treatment with 5 μM DAC and 20 μM curcumin significantly decreased the total DNMT activity as compared with treatment with 20 μM curcumin only (Fig. 4). The protein expression of DNMT3a was also regulated, being significantly decreased by combined treatment with 5 μM DAC and 20 μM curcumin; the DNMT3b protein expression was also lower with combined treatment in comparison to treatment with 5 μM DAC or 20 μM curcumin alone (Fig. 5A,B).

### Effects of curcumin alone and in combination with DAC on the protein expression level of β-catenin and expressions of downstream genes of the Wnt/β-catenin signaling pathway

**Figure 6:**
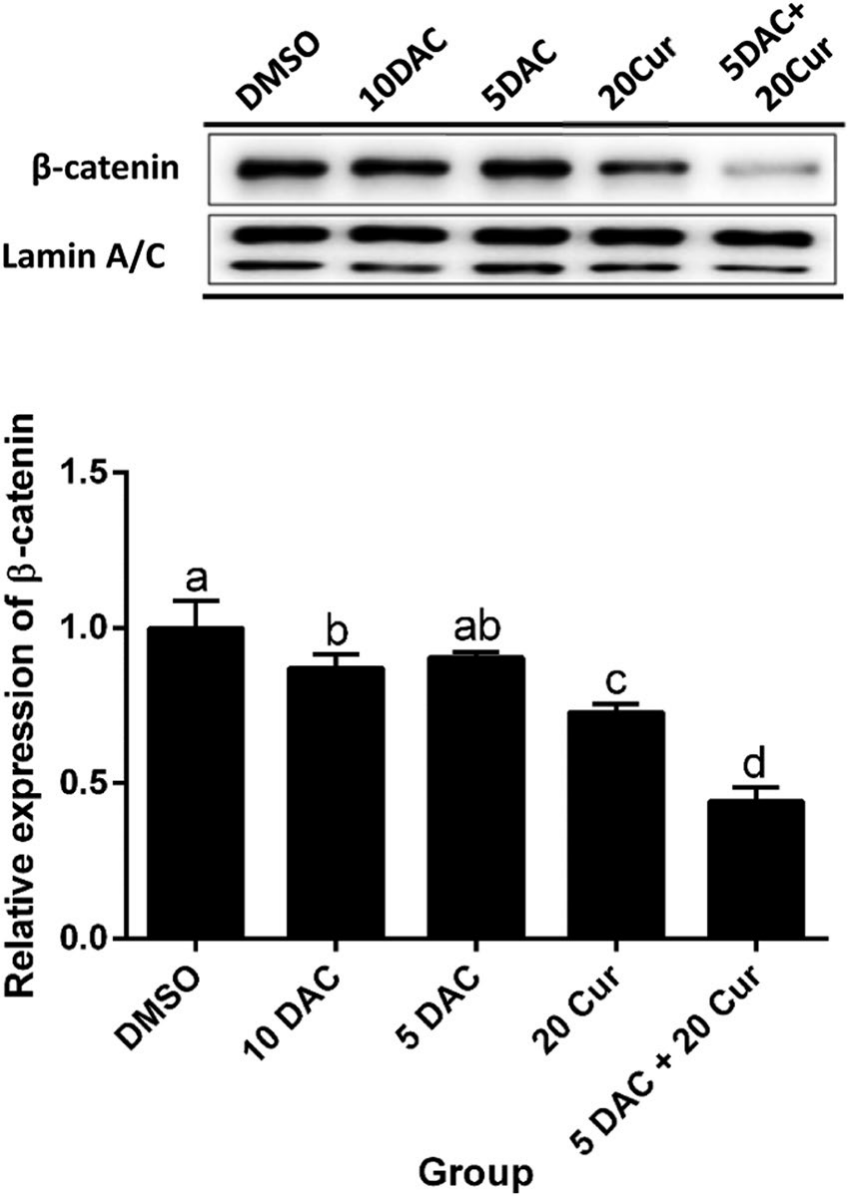
Effects of curcumin alone and in combination with DAC for 96 hours on the protein expression level of β-catenin in SKOV3 ovarian cancer cells. (**A**) Immunoblots of β-catenin protein. (**B**) Densitometric analysis of β-catenin protein. 10 DAC, 10 μM DAC; 5 DAC, 5 μM DAC; 20 Cur, 20 μM curcumin. Data are expressed as means ± SD of triplicate experiments. ^a,b,c,d^Bars without the same letters on top are statistically significant among treatments when compared to each other, as determined by one-way ANOVA followed by Duncan’s *post hoc* test (*p* < 0.05).

**Figure 7:**
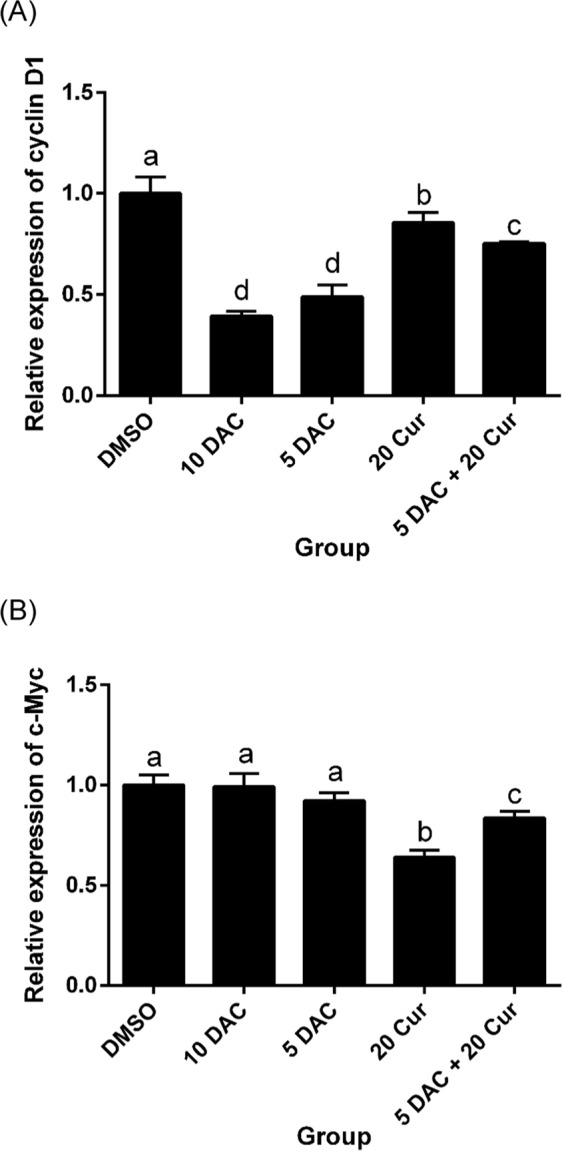
Effects of curcumin alone and in combination with DAC for 96 hours on the mRNA expressions of downstream genes of the Wnt/β-catenin signaling pathway in SKOV3 ovarian cancer cells. mRNA expression levels of cyclin D1 (**A**) and c-Myc (**B**). 10 DAC, 10 μM DAC; 5 DAC, 5 μM DAC; 20 Cur, 20 μM curcumin. Data are expressed as means ± SD of triplicate experiments. ^a,b,c,d^Bars without the same letters on top are statistically significant among treatments when compared to each other, as determined by one-way ANOVA followed by Duncan’s *post hoc* test (*p* < 0.05).

β-catenin is a key nuclear factor in the canonical Wnt signaling pathway in the nucleus. Imbalance in signaling may lead to the triggering of Wnt-specific downstream genes, such as Cyclin D1 and c-Myc. β-catenin in the nucleus was significantly decreased by 10 μM DAC, 20 μM curcumin, and a combination of both, 5 μM DAC and 20 μM curcumin reducing the protein expression of β-catenin by more than half (Fig. 6). The expression levels of Wnt/β-catenin signaling pathway downstream genes Cyclin D1 and c-Myc were reduced by both DAC and curcumin treatment, and combined treatment with 5 μM DAC and 20 μM curcumin also decreased the expressions of both cyclin D1 and c-Myc. The inhibition effect on cyclin D1 expression of 5 and 10 μM DAC was stronger than that of 20 μM curcumin, while the expression of c-Myc was lowered by 5 and 10 μM DAC treatment to a greater degree than by treatment with 20 μM curcumin (Fig. 7A,B).

### Effects of curcumin alone and in combination with DAC on cell migration and the mRNA expressions of EMT marker genes

**Figure 8:**
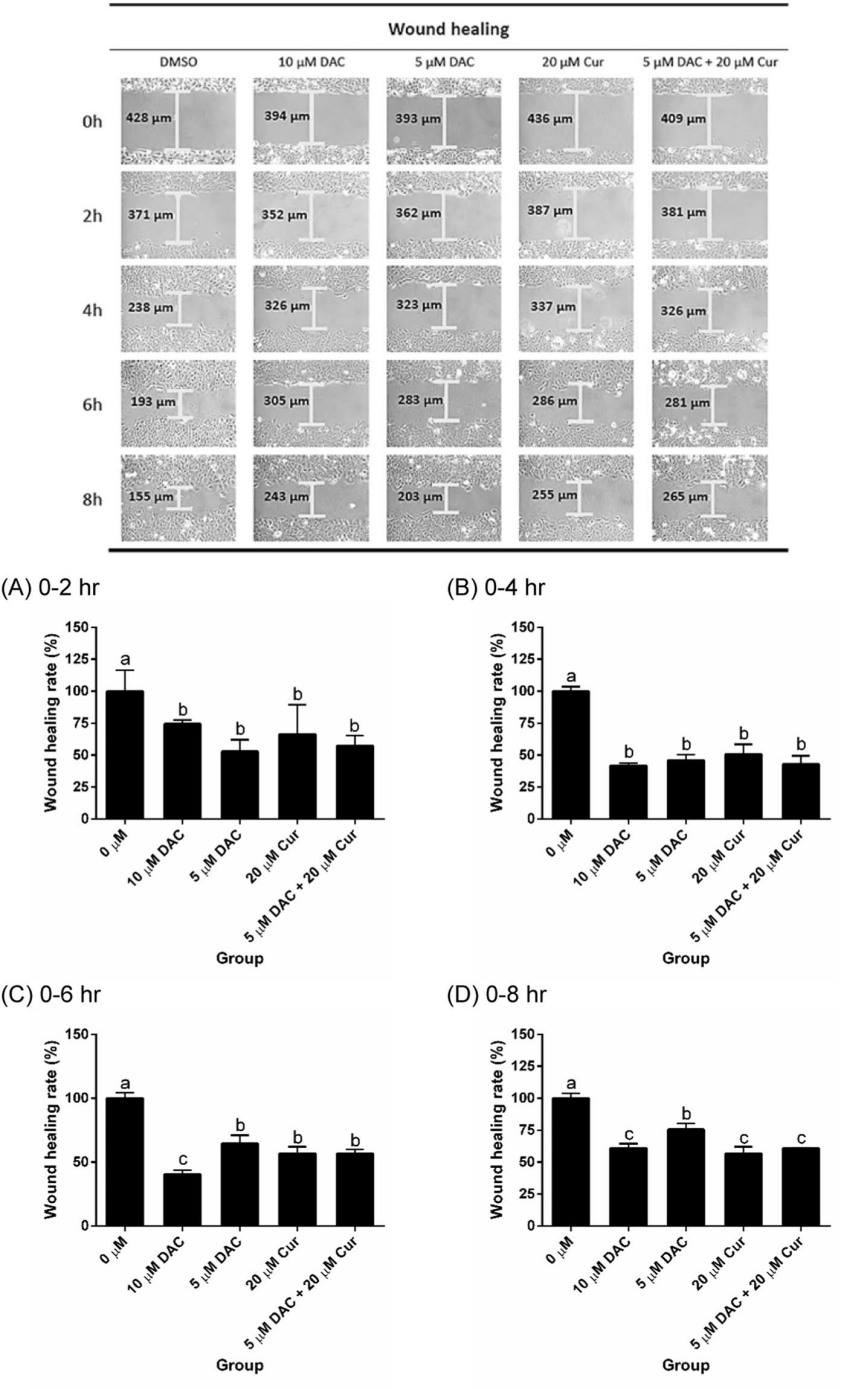
Effects of curcumin alone and in combination with DAC for 96 hours on the cell migration of SKOV3 ovarian cancer cells. (**A**) 0–2 hours; (**B**) 0–4 hours; (**C**) 0–6 hours; (**D**) 0–8 hours. Data are expressed as means ± SD of triplicate experiments. ^a,b,c,d^Bars without the same letters on top are statistically significant among treatments when compared to each other, as determined by one-way ANOVA followed by Duncan’s *post hoc* test (*p* < 0.05).

**Figure 9:**
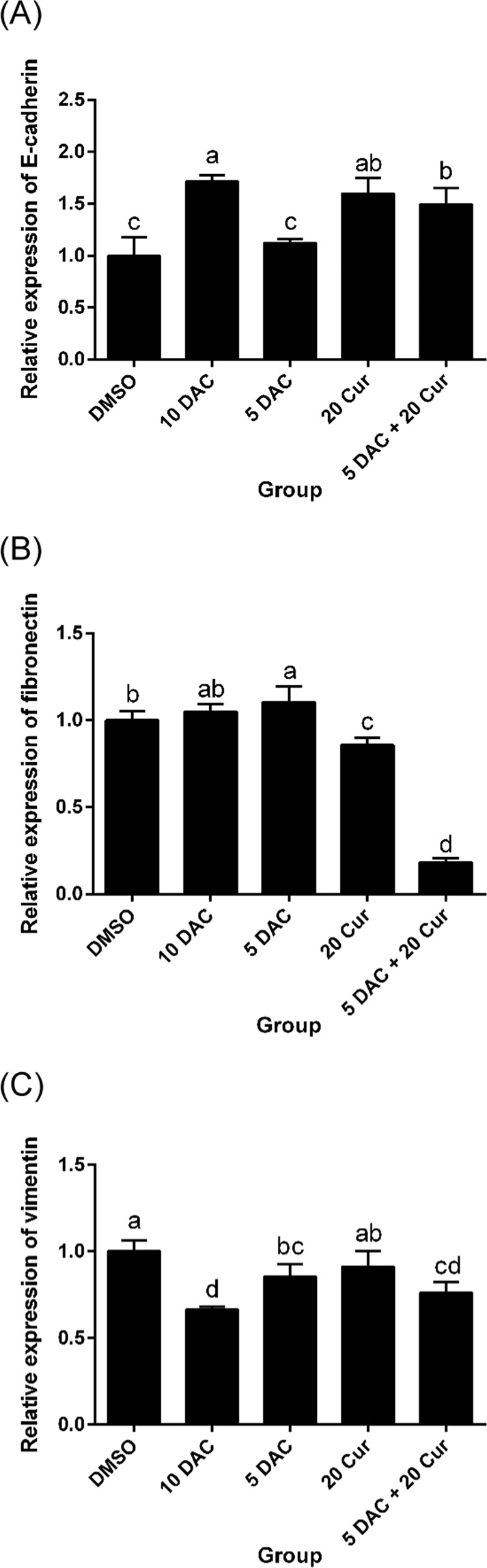
Effects of curcumin alone and in combination with DAC for 96 hours on mRNA expressions of EMT marker genes in SKOV3 ovarian cancer cells. mRNA expression levels of E-cadherin (**A**), fibronectin (**B**), vimentin (**C**). 10 DAC, 10 μM DAC; 5 DAC, 5 μM DAC; 20 Cur, 20 μM curcumin. Data are expressed as means ± SD of triplicate experiments. ^a,b,c,d^Bars without the same letters on top are statistically significant among treatments when compared to each other, as determined by one-way ANOVA followed by Duncan’s *post hoc* test (*p* < 0.05).

We investigated SKOV3 cell migration after treatment with DAC, curcumin, and a combination of both. The results showed that SKOV3 cell migration was inhibited by DAC and curcumin. There was no difference in terms of the effect on cell migration between single treatments and combined treatment with 5 μM DAC and 20 μM curcumin (Fig. 8). The EMT marker genes of E-cadherin, which is an epithelial marker, were up-regulated by treatment with 10 μM DAC and 20 μM curcumin. Combined treatment with 5 μM DAC and 20 μM curcumin increased the expression of E-cadherin to a greater degree than single treatment with 5 μM DAC (Fig. 9A). The expressions of mesenchymal markers such as fibronectin and vimentin were significantly reduced by combined treatment with 5 μM DAC and 20 μM curcumin (Fig. 9B,C). This means that combined treatment with both DAC and curcumin effectively inhibits the EMT process in SKOV3 ovarian cancer cells.

## Discussion

The Wnt signaling pathway is involved in cell proliferation, differentiation and fate determination^39^. However, many studies have demonstrated that the Wnt/β-catenin signaling pathway is involved in carcinogenesis^40^, and the signaling components are often abnormally expressed in many cancers, including ovarian cancer^41^. A meta-analysis study showed that SFRP methylation may contribute to carcinogenesis especially in hepatocellular carcinoma and colorectal cancer cancers^42,43^, in our previous study also showed that epigenetic silencing of SFRP5 may leads to ovarian cancer by oncogenic activation of the Wnt pathway^23^. The clinical limitations of ovarian cancer are late diagnosis and ineffective chemotherapy cause the number of deaths^7,44^. Therefore, with increasing awareness of the critical need for more effective and less toxic treatment options for cancer patients, combined treatment of chemotherapy and natural compounds with inherent characteristics that could be a therapeutic strategy.

In our study, SFRP5 was fully methylated and genes silenced in SKOV3 ovarian cancer cells. We used a lower dose of DAC and treated cells with a combination of 5 μM DAC and 20 μM curcumin, following which SFRP5 was slightly expressed. This result showed that DAC alone and in combination with curcumin inhibits DNA methylation. DAC was used as a DNMT inhibitor, the effects of which have been demonstrated in previous research^45^. Non-nucleoside inhibitors can inhibit DNMT by blocking the active sites of enzymes or acting as a DNMT target sequence, such as epigallocatechin-3-gallate (EGCG) and procaine, respectively^46,47^. Studies have reported that curcumin may block DNMT1 by covalently binding to the catalytic thiolate site^48^; however, another study indicated that curcumin has only slight activity as a DNMT inhibitor^49^.

Although the mechanism of DNMT inhibition by curcumin is not well-known, our study results showed that curcumin inhibited the total DNMT activity and DNMT3a protein expression. In addition, the inhibition of DNMT upon combined treatment with 5 μM DAC and 20 μM curcumin was stronger than that caused by single treatment with either DAC or curcumin. We suggest that curcumin may exert adjuvant effects in ovarian cancer chemotherapy. Curcumin possesses chemopreventive activity to inhibit carcinogenesis, including suppressing the initiation and proliferation of cancer cells^50^. In addition, curcumin exerts a chemosensitization effect, in terms of improving and enhancing the efficacy of anti-neoplastic agents by restraining pathways that lead to resistance to therapy^51^. In the present study, we found that combined treatment with 5 μM DAC and 20 μM curcumin significantly inhibited β-catenin protein expression, and the inhibitory effect was stronger than the effects of single treatments. In addition, downstream genes cyclin D1 and c-Myc were down-regulated, leading to suppression of tumorigenesis via inhibition of colony formation in SKOV3 cells. Previous study has indicated that curcumin is a good inhibitor of the Wnt/β-catenin signaling pathway in colon, gastric, intestinal and prostate cancer cell lines^52^. A study by Liu *et al*.^53^ investigated treatment with the chemotherapy drug paclitaxel combined with curcumin in multidrug-resistant (MDR) A2780 ovarian cancer cells, and found that curcumin had the ability to overcome MDR cancer cells by increasing the paclitaxel concentration and improving the anti-cancer activity. Accordingly, treatment with a combination of curcumin and DAC may improve the effects of therapy on SKOV3 ovarian cancer cells. The regulatory effects of DAC and curcumin on cell migration were also investigated, and the results showed that either single or combination treatment with DAC and curcumin inhibited SKOV3 cell migration. One crucial EMT-associated gene, vimentin, is also a target gene of the Wnt/β-catenin signaling pathway^54^. Our results showed that not only vimentin, but also fibronectin, were down-regulated, whereas E-cadherin was up-regulated, by combined treatment with 5 μM DAC and 20 μM curcumin.

In general, the results illustrated the adjuvant therapy effects of curcumin with DAC in terms of inhibiting cell proliferation and migration through DNA methylation modification and Wnt/β-catenin signaling pathway regulation in ovarian cancer cells. This treatment may be used as a strategy for chemotherapy, combining a natural compound and a drug to reduce the dosage of anti-neoplastic agents, which lowers the risk of side effects and enhances the efficacy of treatment.

## Conclusion

The results of the present study suggested that SFRP5 methylation may be associated with cell proliferation, colony formation and migration in ovarian cancer through Wnt/β-catenin signaling pathway. Combination treatment with 5 μM DAC and 20 μM curcumin inhibited the malignant phenotype, improved SFRP5 re-expression by restraining DNMT, decreasing DNA methylation and inhibiting the Wnt pathway. In addition, cyclin D1 and c-Myc, the downstream genes of Wnt/β-catenin signaling pathway, and the EMT markers were also suppressed by this treatment. Our data also support the idea that curcumin may enhance the effect of chemotherapy, be a potential natural DNMT inhibitor and a novel epigenetic therapy for ovarian cancer.

## Methods

### Cell culture

Ovarian cancer cell line SKOV3 (ATCC HTB-77) was obtained from Dr. Lin (National Defense Medical Center, Taipei City, Taiwan). SKOV3 cells were cultured in RPMI 1640 (Gibco) medium containing 1% non-essential amino acids (Gibco^TM^), 1% sodium pyruvate (Gibco), 10% fetal bovine serum (FBS) and 0.2% penicillin/streptomycin (Biological Industries), and maintained at 37 °C in a humidified atmosphere containing 5% CO_2_.

### Chemical compounds

Curcumin and 5-aza-2′-deoxycytidine (DAC) were purchased from Sigma-Aldrich in Taiwan, dissolved in dimethyl sulfoxide (DMSO), and stored in the dark at −20 °C. SKOV3 cells were treated with curcumin, DAC, or a combination of both, and incubated for 96 hours.

### Cell viability

A CellTiter 96 AQueous One Solution Cell Proliferation assay (Promega) was used to determine the effects of curcumin alone and in combination with DAC on SKOV3 cell viability. SKOV3 cells were seeded onto a 96-well plate (100 μL; 1.5 × 10^3^/well) and incubated for 24 hours. The cells were subsequently exposed to single or combined treatment with curcumin and DAC at different concentrations and incubated for a further 96 hours. Control group cells were treated with DMSO. The cells were then treated with 20 μL MTS reagent and 100 μL medium and incubated for an additional 2 hours. The 96-well plate was read at 490 nm in a VICToR Multilabel Plate Reader (PerkinElmer).

### RNA extraction and RT-PCR

An RNeasy Plus Mini Kit (QIAGEN) was used to isolate the RNA of SKOV3 cells. The concentration of RNA extracted was quantified using NanoDrop 2000 (ThermoFisher) and reverse-transcribed to cDNA using SuperScript® III Reverse Transcriptase (Invitrogen). The target genes of cDNA were amplified by PCR (Biometra) in a 35-cycle reaction. The PCR conditions were: 95 °C for 30 sec, 60 °C for 30 sec, and 72 °C for 30 sec.

### Quantitative real-time PCR (qRT-PCR)

The expression levels of target genes were detected by qRT-PCR using a LightCycler 480 system (Roche) in a 45-cycle process. The PCR conditions were: 95 °C for 30 sec, 60 °C for 30 sec, and 72 °C for 30 sec. The total reaction proceeded in 45 cycles. GAPDH was used as an internal control. The primers were purchased from Genomics (Taiwan), and the sequences are listed in Table 1.

**Table 1:** Gene sequences of primers.

Gene	Forward sequence	Reverse sequence
**MS-PCR**		
SFRP5-M	AAGATTTGGCGTTGGGCGGGACGTTC	ACTCCAACCCGAACCTCGCCGTACG
SFRP5-U	GTAAGATTTGGTGTTGGGTGGGATGTTT	AAAACTCCAACCCAAACCTCACCATACA
**RT-PCR or qRT-PCR**		
SFRP5	CAGATGTGCTCCAGTGACTTTG	AGAAGAAAGGGTAGTAGAGGGAG
CyclinD1	GAACAAACAGATCATCCGCAAAC	GCGGTAGTAGGACAGGAAGTTG
c-Myc	GGCAAAAGGTCAGAGTCTGG	GTGCATTTTCGGTTGTTGC
E-cadherin	CCCACCACGTACAAGGGTC	ATGCCATCGTTGTTCACTGGA
Vimentin	GAACGCCAGATGCGTGAAATG	CCAGAGGGAGTGAATCCAGATTA
Fibronectin	GAAGCCGAGGTTTTAACTGC	ACCCACTCGGTAAGTGTTCC
GAPDH	GCTTCACATACCTTTGGAGCTTC	TCTCTTCCTCTTGTGCTCTTG

### DNA extraction and MS-PCR

DNA was extracted from SKOV3 cells using a DNeasy® Blood & Tissue Kit (QIAGEN) and quantified using NanoDrop 2000 (ThermoFisher). In order to complete the bisulfite conversion of DNA, we used an EZ DNA methylation^TM^ Kit (Zymo Reserch), and further detected the methylation status of SFRP5 by MS-PCR in a 35-cycle reaction. The PCR conditions were: 95 °C for 30 sec, 60 °C for 30 sec, and 72 °C for 30 sec. The primers were purchased from Genomics (Taiwan), and the sequences are listed in Table 1.

### Total protein and nuclear protein extraction

Total protein was extracted from SKOV3 cells using RIPA buffer (ThermoFisher) with 20X proteinase inhibitor (Roche). The supernatant was collected after centrifugation (14,000 × g, 4 °C) for 30 minutes. NE-PER Nuclear and Cytoplasmic Extraction Reagents (Thermo Fisher) were used to isolate nuclear protein from SKOV3 cells. After centrifuge at 14,000 × g at 4 °C for 10 minutes, the supernatant was collected and stored at −80 °C. The concentrations of extracted proteins were analyzed using a BCA protein assay kit (Pierce Biotechnology).

### Western blotting

The target protein expression levels were analyzed using western blotting. Protein samples were loaded onto SDS-PAGE gel (5% stacking gel and 10% separating gel) in tris-glycine running buffer (OmicsBio) and separated by electrophoresis at 100 V. Subsequently, the proteins on the gel were transferred to polyvinylidene fluoride (PVDF) membrane (Millipore) using the Bio-rad system at 100 V for 90 minutes. The membrane was then blocked in 5% skimmed milk and shaken for 1 hour. Primary antibody incubation was carried out at 4 °C with shaking overnight. After washing three times with 3 mL of TBST buffer (tris-buffered saline solution with 0.1% Tween 20), the membrane was probed with secondary antibodies for 1 hour at room temperature, followed by washing three times. The immunoreactive bands of target proteins were then detected using ECL (Bio-Rad) and SPOT Xplorer (BioVision). The intensities of bands were quantified using Image J software (National Institutes of Health, USA). Detailed information regarding the antibodies used for western blotting is presented in Table 2.

**Table 2:** Antibodies used in the study.

Antibody	Dilution ratio	Brand	Cat. No.
**Primary antibody**			
DNMT1	1:1000	Abcam	Ab13537
DNMT3a	1:1000	Santa Cruz	Sc-20703
DNMT3b	1:1000	Novus	52A1018
β-catenin	1:3000	BD Bioscience	610153
Lamin A/C	1:3000	BD Bioscience	612162
β-actin	1:10000	Sigma	A5316
**Secondary antibody**			
Gout anti- mouse IgG-HRP	1:5000	Santa Cruz	Sc-2005
Goat anti- rabbit IgG-HRP	1:4000	Santa Cruz	Sc-2004

### Total activity of DNMT

An EpiQuik DNMT Activity/Inhibition Assay Ultra Colorimetric Kit (Epigentek) was used to detect the total activity of DNMT in SKOV3 cells. In this assay, DNMT substrate was prepared and stably coated onto the microplate wells of the kit. Protein samples were added to each well of the microplate and incubated with substrate for 1.5 hours. The capture antibody was added to each well after washing each well three times with washing buffer, following which the detection antibody and enhancer reagents were added to the wells. Signal detection of the microplate samples was performed using a plate reader (PerkinElmer) at OD 450 nm and calculated using the following formula:

$${\rm{DNMT}}\,{\rm{activity}}\,(\mathrm{OD}/{\rm{h}}/\mathrm{mg})=\frac{(\mathrm{Sample}\,\mathrm{OD}\,-{\rm{Blank}}\,\mathrm{OD})}{(\mathrm{Protein}\,\mathrm{amount}\,({\rm{\mu }}{\rm{g}})\times \mathrm{hour})}\times 1000$$

### Colony-formation assay

The 1.5 mL of 0.5% agarose were prepared as a base layer in a 6-well plate and 1.5 × 10^4^ SKOV3 cells were counted and added, followed by re-suspension in 1.5 mL of 0.35% agarose for seeding in the semi-solid medium. Different doses of curcumin, DAC, and curcumin and DAC combined were mixed with medium and added to each well. The 6-well plate was then incubated in an incubator (37 °C, 5% CO_2_) for four weeks. The 6-well plate was subsequently stained with 2 mL of 0.005% crystal violet for 30 minutes after drying at room temperature overnight. The plate was washed three times and then air-dried. Finally, the number of colonies was quantified.

### Cell migration

SKOV3 cell migration was analyzed using a wound-healing assay. Culture insert (ibidi) was set into a 12-well plate, following which SKOV3 cells were counted and seeded (8 × 10^5^) into each well containing the culture insert with 70 μL of medium and incubated for 24 hours. Suction was used to draw out the medium in the insert, and the insert was gently removed using sterile tweezers, followed by the addition of 2 mL of medium to each well of the plate. Finally, cell migration was observed for 0–8 hours.

### Statistical analysis

All of the results are presented as the mean ± standard deviation (SD) of triplicate experiments. Data were analyzed using Statistical Analysis System (SAS) software, version 9.4, and the different letters indicate statistically-significant differences between different treatments, as determined by one-way analysis of variance (ANOVA) and Duncan’s *post hoc* test (*p* < 0.05).
